# Fzr regulates silk gland growth by promoting endoreplication and protein synthesis in the silkworm

**DOI:** 10.1371/journal.pgen.1010602

**Published:** 2023-01-18

**Authors:** Wenliang Qian, Hao Li, Xing Zhang, Yaohao Tang, Dongqin Yuan, Zhu Huang, Daojun Cheng

**Affiliations:** 1 State Key Laboratory of Silkworm Genome Biology, Biological Science Research Center, Southwest University, Chongqing, China; 2 Chongqing Key Laboratory of Sericultural Science, Chongqing engineering and technology research center for novel silk materials, Southwest University, Chongqing, China; University of Kentucky, UNITED STATES

## Abstract

Silkworm silk gland cells undergo endoreplicating cycle and rapid growth during the larval period, and synthesize massive silk proteins for silk production. In this study, we demonstrated that a binary transgenic CRISPR/Cas9 approach-mediated *Fzr* mutation in silkworm posterior silk gland (PSG) cells caused an arrest of silk gland growth and a decrease in silk production. Mechanistically, PSG-specific *Fzr* mutation blocked endoreplication progression by inducing an expression dysregulation of several cyclin proteins and DNA replication-related regulators. Moreover, based on label-free quantitative proteome analysis, we showed in PSG cells that *Fzr* mutation-induced decrease in the levels of cyclin proteins and silk proteins was likely due to an inhibition of the ribosome biogenesis pathway associated with mRNA translation, and/or an enhance of the ubiquitin-mediated protein degradation pathway. Rbin-1 inhibitor-mediated blocking of ribosomal biogenesis pathway decreased DNA replication in PSG cells and silk production. Altogether, our results reveal that Fzr positively regulates PSG growth and silk production in silkworm by promoting endoreplication and protein synthesis in PSG cells.

## Author summary

Increasing evidence in mammalian and *Drosophila* have revealed that Fzr is essential for mitotic-to-endocycle transition and endoreplication progression. How Fzr functions in endoreplicating silkworm silk glands remains unclear. Based on CRISPR/Cas9-mediated *Fzr* mutation analysis in silkworm PSG cells, we demonstrate that Fzr plays key roles in the control of gland size, silk protein synthesis, and DNA replication during silk gland growth. We mechanistically uncover that Fzr not only positively regulates the ribosome pathway to affect the translation of cell cycle regulators and silk proteins but also downregulates the ubiquitin-mediated protein degradation pathway, therefore maintaining the endoreplication of silk gland cells, gland growth, and silk production.

## Introduction

The silkworm (*Bombyx mori*) is an economically important insect that synthesize silk proteins for silk production in silk glands. Silkworm silk gland consists of three parts, namely anterior silk gland (ASG), middle silk gland (MSG), and posterior silk gland (PSG). During the early embryonic stage, silk gland cells undergo about 10 rounds of mitosis to form certain cell numbers [1]. But, at the late embryonic stage, silk gland cells enter endoreplication and DNA contents in silk gland cells reach to 300,000–500,000C (C value, namely DNA content in haploid nucleus) following about 17–19 rounds of endoreplicating cycle during the larval stage, forming giant cells and gland [2–4]. The MSG cells mainly synthesizes sericin proteins (e. g. Ser1) and PSG cells mainly produces fibroin proteins (e. g. FibH, FibL, and P25) [5]. These synthesized silk proteins are secreted into the lumen and then form silk fiber for spinning a cocoon during the wandering stage [6].

Endoreplication, also named endocycle, is a special cell cycle throughout plants and animals that undergoes multiple rounds of DNA replication without chromosome segregation or cytokinesis [7]. Previous studies in silkworm have showed that among several cyclin proteins, CycE, but not CycB, is expressed in silk glands of silkworm larvae [4,8]. The endoreplicating cycle in silk glands can be regulated by ecdysone and insulin signaling [9,10]. The overexpression of *Ras*, *Yorkie*, or *Myc* in the PSG promotes endoreplication progression and elevated DNA content, silk protein expression, and silk yield [11–13]. In addition, *laminA/C* mutation in PSG cells decrease nucleus size, DNA content, and silk production, and causes an abnormal gland development [14]. To date, the regulatory mechanism underlying endoreplication in silk gland cells and gland growth are poorly understood.

Increased evidence in *Drosophila* and mammals have confirmed that high expression of the scaffold protein Fzr drives the mitotic-to-endocycle transition and maintains the oscillation of DNA replication during endoreplication [7,15–18]. Previous studies in *Drosophila* demonstrated that loss of Fzr function blocks the mitotic-to-endocycle transition and endoreplication progression in larval salivary gland, larval prothoracic gland, and follicle cells of adult ovary [15,16,19,20]. Ectopic *Fzr* expression in diploid cells, such as follicle cells, wing disc cells, and *Drosophila* S2 cells, induces the endoreplication entrance [15,21–24]. In our previous study, a novel Fzr-H2Bub-Myc signaling cascade has been characterized to regulate endoreplication progression in *Drosophila* salivary glands [23]. However, the function of Fzr in silkworm silk glands with endoreplicating cell cycle remains unclear.

In the present study, we performed a CRISPR/Cas9-mediated mutation of the *Fzr* gene to determine its function in silkworm silk glands. PSG-specific *Fzr* mutation arrested gland growth and decreased silk production. Mechanistically, *Fzr* mutation blocked DNA replication through a dysregulation in cyclin proteins (CycE, CycD, and CycB) and DNA replication-related proteins (mini-chromosome maintenance proteins (MCMs) and Myc). Moreover, *Fzr* mutation-caused downregulation in the ribosome biogenesis pathway probably decreased the translation of cell cycle regulators and silk proteins, but an enhance of the ubiquitin-mediated protein degradation pathway may be associated with an increase in total protein ubiquitination levels. In addition, blocking the ribosome biogenesis pathway inhibited DNA replication in PSG cells and decreased silk production, which phenocopied PSG-specific *Fzr* mutation. These findings provide novel insights into regulatory mechanism underlying endoreplication progression and silk protein synthesis in silkworm silk glands.

## Results

### PSG-specific *Fzr* mutation decreased the size of silk glands

**Fig 1:**
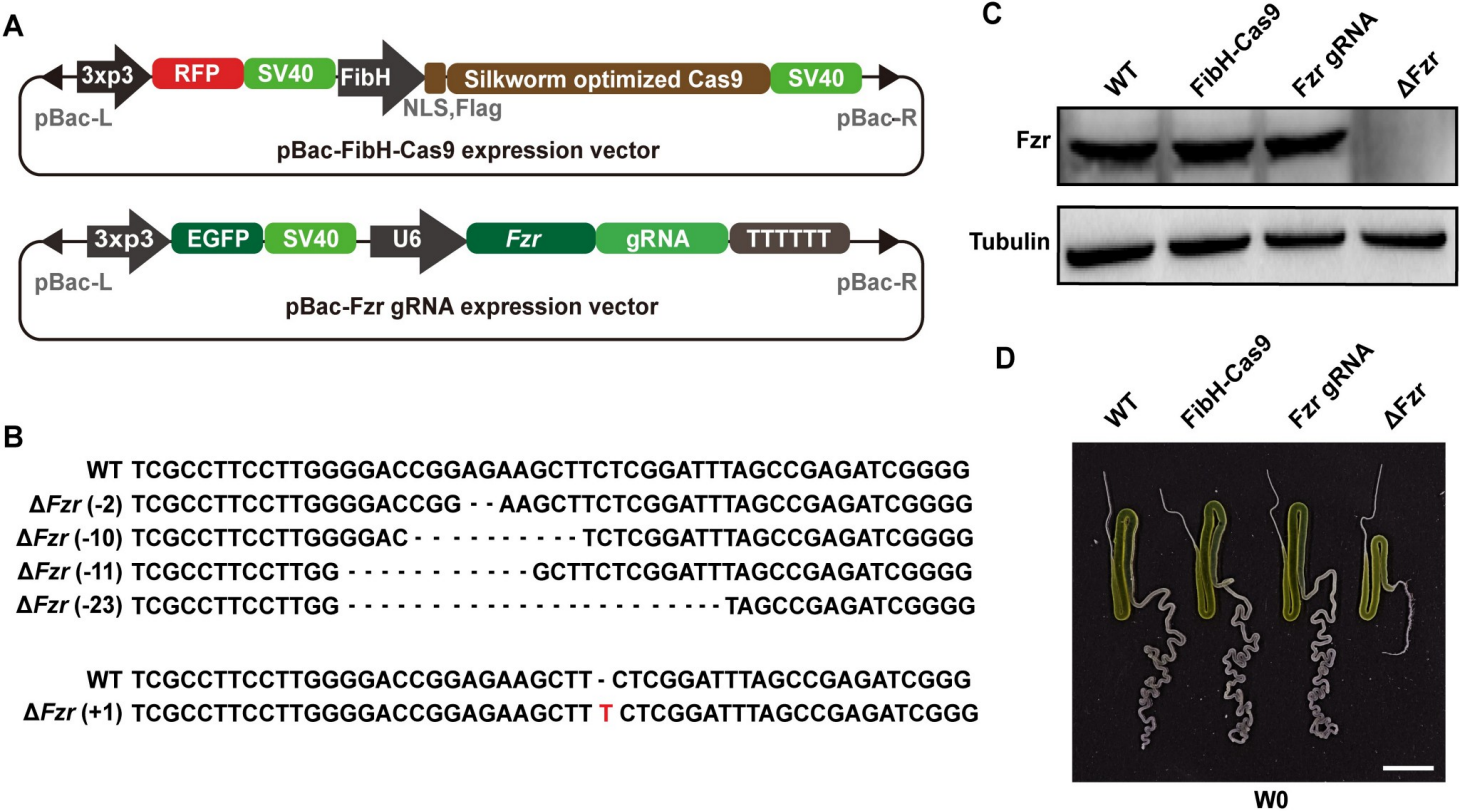
PSG-specific mutation of the *Fzr* gene decreases gland size in silkworm. (**A**) Schematic diagram of the recombinant plasmid used to mutate *Fzr* gene in silkworm PSG. (**B**) Genomic PCR products was sequenced to evaluate CRISPR/Cas9-mediated mutation efficiency. (**C**) Western blotting confirmed that *Fzr* was efficiently mutated in the PSG. (**D**) *Fzr* mutation resulted in a decrease in the PSG size. Scale bar, 1 cm. WT, wild type. W0, just wandering.

To decipher the function of Fzr in silkworm silk glands, we mosically mutated silkworm *Fzr* gene in PSG cells using a binary transgenic CRISPR/Cas9 approach according to previous report [25]. The Cas9 line that was driven by PSG-specific *FibH* promoter and ubiquitous *U6* promoter-driven gRNA line that targets the second exon of the silkworm *Fzr* gene were generated, respectively (Figs 1A and S1A). After crossing the Cas9 line with the gRNA line, different types of *Fzr* mutations could be detected in PSG cells during the fourth larval instar (Fig 1B). Western blotting showed that compared with the lines as the controls, including wild type (WT), *FibH*-Cas9, and *Fzr* gRNA, Fzr protein expression disappeared in the PSG of the progenies from the crossing (Fig 1C), confirming that the *Fzr* gene was efficiently mutated in the PSG.

We analyzed the effects of PSG-specific *Fzr* mutation on gland development. The result showed that compared to controls, the PSG size had no obvious change during the second and third larval instar (S1B and S1C Fig), but was dramatically decreased after the second day of the fourth larval instar (L4D2) following PSG-specific *Fzr* mutation (Figs 1D and S1D–S1E). These results indicated that Fzr was essential for silkworm silk gland growth.

### PSG-specific *Fzr* mutation decreased silk protein synthesis

**Fig 2:**
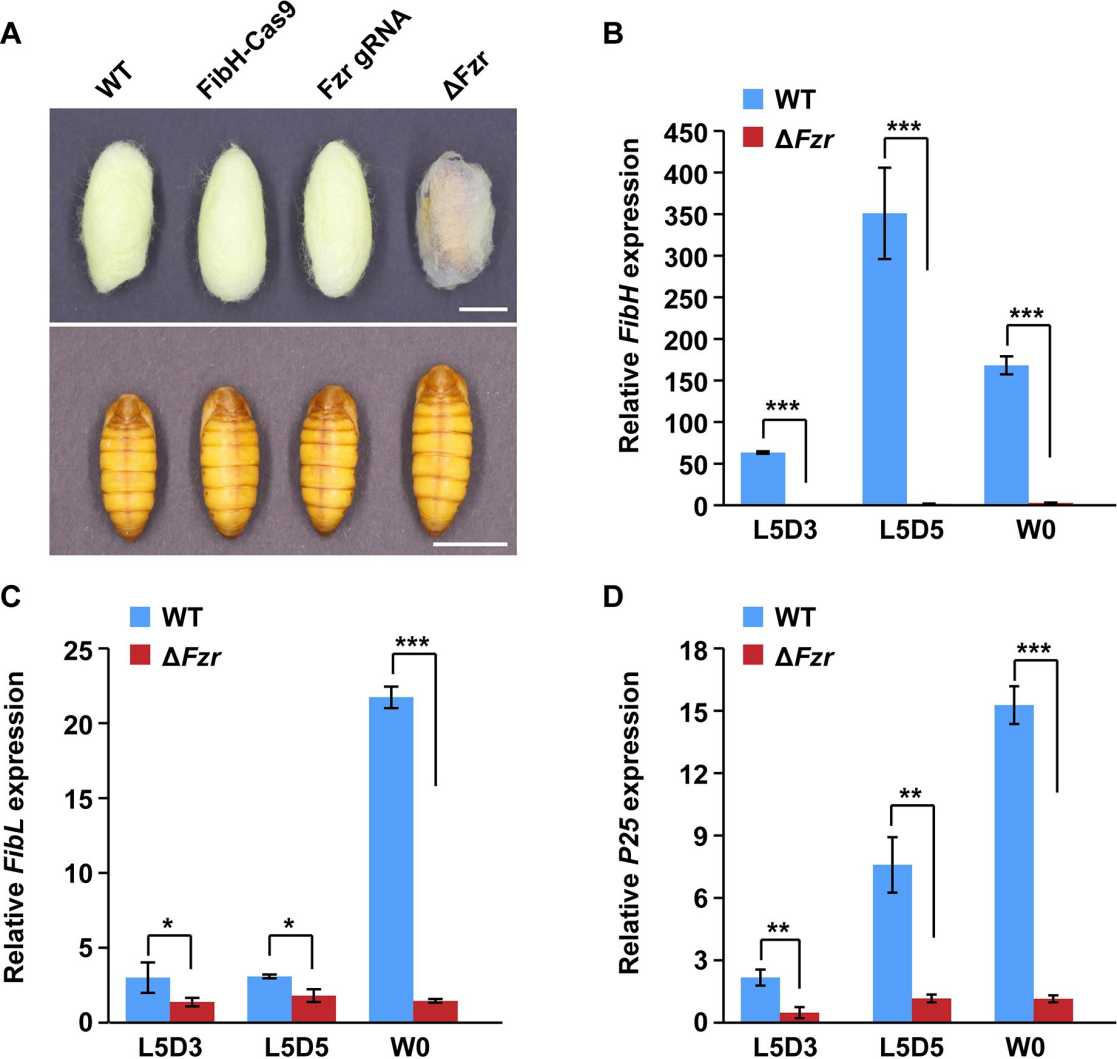
PSG-specific *Fzr* mutation decreases the expression of silk protein genes. (A) *Fzr* mutation in the PSG resulted in thinner cocoon compared with controls. (**B-D**) *Fzr* mutation in the PSG decreased the transcription of silk protein genes, including *FibH* (B), *FibL* (C), and *P25* (D). Values were represented as means ±S.E. (error bars). For the significance test: *P < 0.05, **P < 0.01, and ***P < 0.001 versus the control. Scale bar, 1cm. WT, wild type. L5D3, the third day of the fifth larval instar; L5D5, the fifth day of the fifth larval instar; W0, just wandering.

We next investigated the effects of PSG-specific *Fzr* mutation on silk protein synthesis. As shown in Fig 2A, *Fzr* mutant silkworm larvae produced defective cocoons that were thin and nearly transparent. In addition, the size of *Fzr* mutant pupae was moderately increased following *Fzr* mutation (Fig 2A), which may be associated with nutrient redistribution between silk gland growth and individual growth as described in previous report [26]. Further RT-qPCR experiments demonstrated that during the fifth larval instar, the transcription of three PSG-specific silk protein genes, including *FibH*, *FibL* and *P25*, were remarkably downregulated after PSG-specific *Fzr* mutation (Fig 2B–2D). Notably, *FibH* expression was almost reduced to undetectable level following *Fzr* mutation (Fig 2B). These data revealed that *Fzr* mutation in the PSG decreased silk protein synthesis and silk production.

### PSG-specific *Fzr* mutation inhibited DNA replication in PSG cells

**Fig 3:**
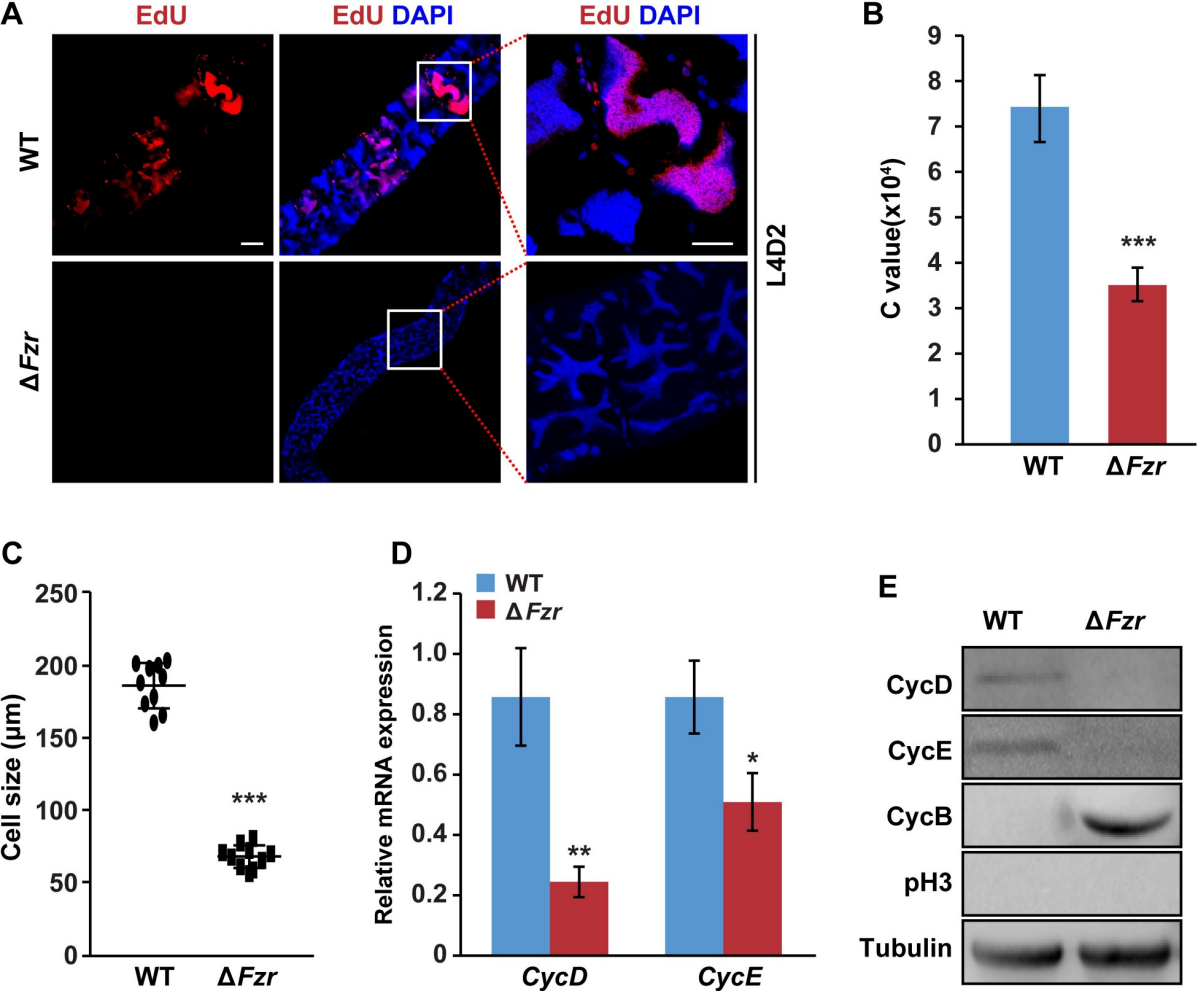
*Fzr* mutation blocks endoreplication in PSG cells. (**A**) EdU staining in silkworm PSG cells. The PSG cells of the control could be strongly stained with EdU, but no EdU signal was detected in PSG cells with *Fzr* mutation. (**B-C**) PSG-specific *Fzr* mutation decreased C value (B) and cell size (C). (**D-E**) RT-qPCR examination and western blotting analysis for the effects of PSG-specific *Fzr* mutation on the expression of cell cycle-related factors. Values were represented as means ±S.E. (error bars). For the significance test: *P < 0.05, **P < 0.01, and ***P < 0.001 versus the control. Scale bar, 100 μm. WT, wild type.

Given that silk gland cells undergo endoreplication during silkworm larval development, we further analyzed whether *Fzr* mutation-caused decrease in silk protein synthesis and gland size were due to a defective in endoreplication progression in PSG cells. EdU staining showed that during the second larval instar, DNA synthesis was undergoing in PSG cells of the controls and the progenies from the crossing of FibH-Cas9 line and *Fzr* gRNA line (S2A Fig). However, DNA synthesis was completely abrogated in PSG cells of *Fzr* mutants compared to controls at L4D2 (Fig 3A), but no change in DNA synthesis was observed in ASG and MSG cells as negative controls (S2B and S2C Fig). Moreover, the DNA content and cell size were remarkably decreased in PSG cells of *Fzr* mutants (Fig 3B and 3C). But, *Fzr* mutation had no effect on the number of PSG cells (S2D Fig). These data revealed that Fzr promoted DNA replication in endoreplicating silk gland cells to define the growth and size of silk glands.

Our previous report outlines a Fzr-H2Bub-Myc signaling cascade during endoreplication progression [23]. Here, RT-PCR and western blotting analysis showed that the loss of Fzr function similarly downregulated H2B ubiquitination and the transcription of *Myc* and *MCMs*, but upregulated *CycB* transcription (S3 Fig), indicating that the Fzr-H2Bub-Myc signaling cascade was also conserved in silk glands. In addition, we further profiled *Fzr* mutation-caused expression changes of other factors involved in cell cycle progression. RT-qPCR analysis revealed that *Fzr* mutation downregulated the mRNA level of *CycD* and *CycE* genes that are related to the G1/S transition (Fig 3D). Western blotting assay found that *Fzr* mutation led to the loss of CycD and CycE but an accumulation of CycB protein in PSG cells, but pH3 signal as a maker of mitotic cells was undetectable in PSG cells of both WT and *Fzr* mutant (Fig 3E). Altogether, these data indicated that although PSG-specific *Fzr* mutation blocked endoreplication progression in PSG cells by inducing an expression dysregulation of several cell cycle-related regulators, the cells could not enter mitotic cycle and arrested at G2/M phase.

### *Fzr* mutation disrupted protein synthesis and degradation pathways

**Fig 4:**
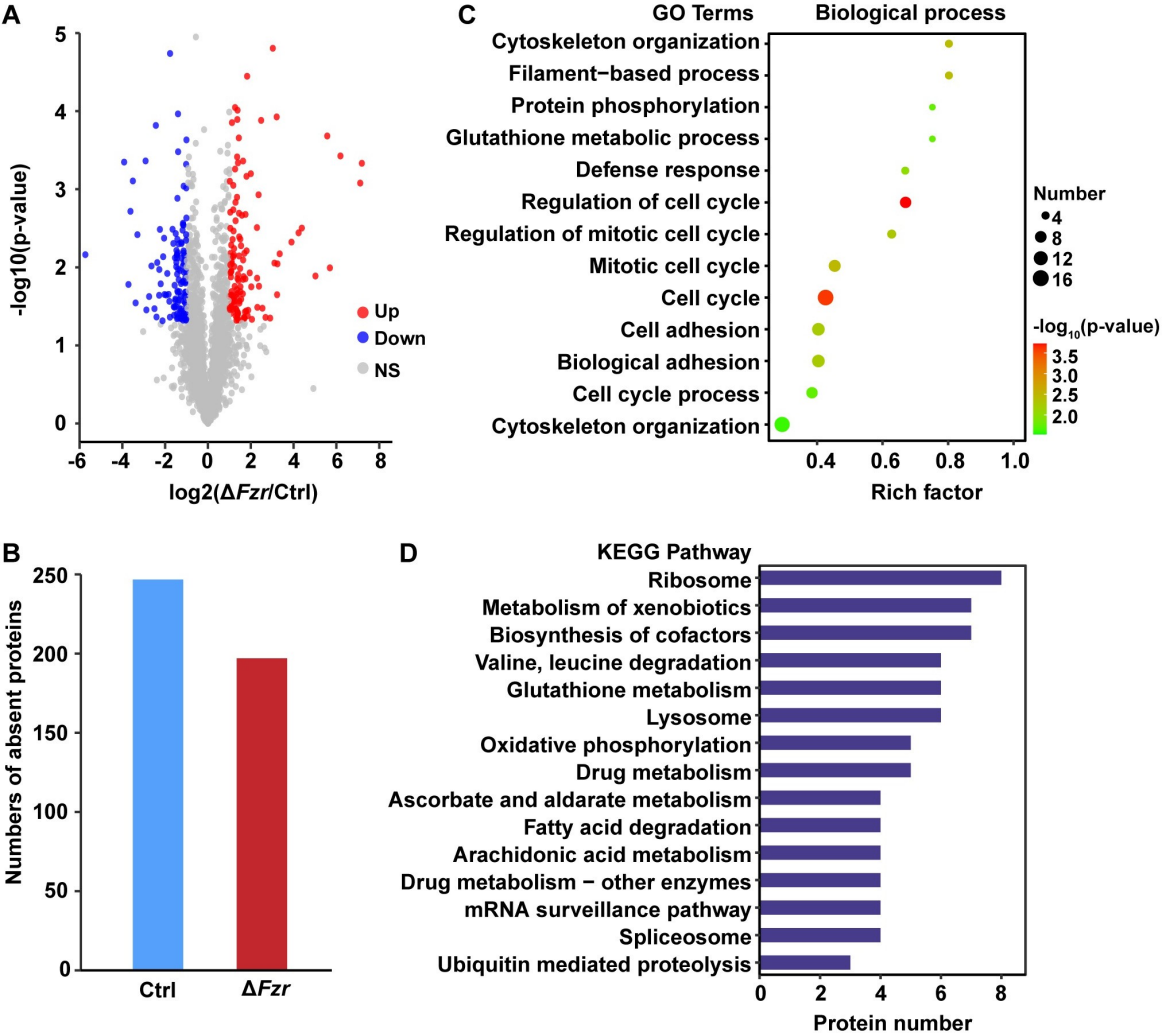
Label-free quantitative proteomic analysis of *Fzr* mutation-caused proteome change in the PSG. (**A**) The volcano plots of the quantified proteins. Red points represented upregulated proteins and blue points represented downregulated proteins. DEPs were identified with a regular criterion of *P* value < 0.05 and log2 fold change (log2FC) ≥ 1.0. (**B**) DEPs were present or absent following *Fzr* mutation in the PSG. (**C**) GO annotation of DEPs in biological process class. Size of dots represented numbers of enriched proteins; Color of dots represented the GO cluster with a highlighted representative term. (**D**) The enriched KEGG pathways.

To decipher the regulation network underlying *Fzr*-mediated endoreplication and silk gland growth in silkworm, we further analyzed the changes of PSG proteome following PSG-specific *Fzr* mutation. LC-MS/MS quantitative proteomics in the PSG of both WT and *Fzr* mutant identified a total of 995549 spectrums, in which 181799 peptide spectrum matches (PSMs) were assigned to 30507 peptides (including 29122 unique peptides) at 1% False-Discovery Rate (FDR) (S4 Fig and S1 and S2 Tables). These identified peptides correspond to 4032 proteins and 3486 of which could be quantified (S4 Fig and S1 and S2 Tables). In addition, comparative analysis identified 693 differentially expressed proteins (DEPs) in the PSG between WT and *Fzr* mutant, including 247 DEPs that fit a regular criterion (p value < 0.05 and log2 fold change (log2FC) ≥ 1.0) and 446 DEPs that were present or absent after PSG-specific *Fzr* mutation (Figs 4A–4B and S5A and S3 Table). Notably, compared with WT, 325 DEPs and 368 DEPs were upregulated and downregulated following *Fzr* mutation, respectively (S4A Fig and S3 Table).

**Fig 5:**
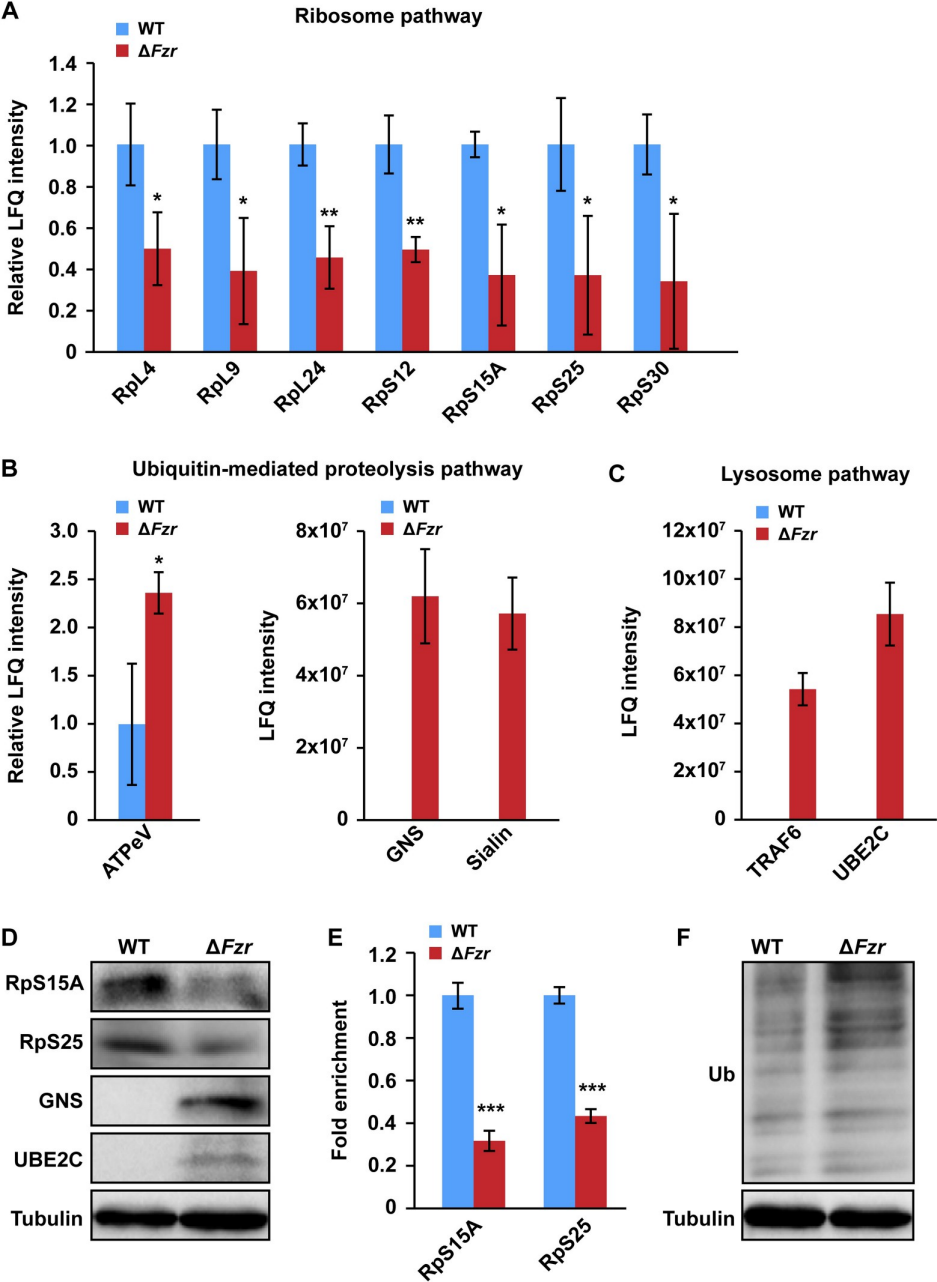
Fzr regulation on protein synthesis and degradation. (**A**) All DEPs in Ribosome pathway were downregulated in PSG cells following PSG-specific *Fzr* mutation. (**B-C**) DEPs in the ubiquitin-mediated proteolysis pathway (B) and lysosome pathway (C) were downregulated by *Fzr* mutation in the PSG. (**D**) Western blotting showed that RpS15A and RpS25 were downregulated, but GNS and UBE2C were elevated following *Fzr* mutation. (**E**) Translating ribosome affinity purification (TRAP) analysis showed that *Fzr* mutation inhibited mRNA translation in PSG cells. (**F**) *Fzr* mutation elevated protein ubiquitination in PSG cells. Values were represented as means ±S.E. (error bars). For the significance test: *P < 0.05, **P < 0.01, and ***P < 0.001 versus the control. WT, wild type.

Gene Ontology (GO) enrichment analysis showed that the DEPs that were associated with cell cycle, cell adhesion, and cytoskeleton organization were enriched in biological process (Fig 4C and S4 Table), and the DEPs showing the capacities of cytoskeletal protein binding, microtubule binding, or tubulin binding were enriched in molecular function class (S5B Fig and S4 Table). Moreover, a Kyoto Encyclopedia of Genes and Genomes (KEGG) analysis revealed that most DEPs were involved in ribosome, amino acid metabolism, ubiquitin-mediated proteolysis, lysosome, and oxidative phosphorylation (Fig 4D and S5 Table). As shown in Fig 5 and S5 Table, all DEPs that function as ribosome constituents were downregulated (Fig 5A), indicating that ribosomal biogenesis and protein synthesis in silkworm PSG were decreased following *Fzr* mutation. On the contrary, the DEPs involved in ubiquitin-mediated proteolysis and lysosome pathways, two pathways related to protein degradation, were upregulated (Fig 5B and 5C), suggesting that *Fzr* mutation may promote protein degradation in the PSG.

We further performed western blotting experiments to investigate the expression changes of several differential proteins from proteomic analysis, including ribosomal protein S15a (RpS15A) and ribosomal protein S25 (RpS25) in the ribosome pathway as well as glucosamine-6-sulfatase (GNS) and ubiquitin-conjugating enzyme E2 C (UBE2C) in the ubiquitin-mediated lysosomal pathway. Consistently, the results showed that RpS15A and RpS25 were downregulated following *Fzr* mutation, but GNS and UBE2C were elevated (Fig 5D). TRAP (translating ribosome affinity purification) analysis showed that compared to WT, entire mRNA translation in PSG cells of *Fzr* mutant was highly inhibited (Fig 5E). Subsequent RT-qPCR assay revealed that the mRNA translation of three PSG-specific silk protein genes (*FibH*, *FibL*, and *P25*) and two cyclin protein genes (*CycD* and *CycE*) were obviously downregulated after *Fzr* mutation (S6A–S6E Fig). Moreover, an anti-ubiquitin (Ub) antibody-based western blotting assay showed that *Fzr* mutation enhanced the ubiquitination level of total proteins (Fig 5F). Altogether, we speculated that Fzr regulated endoreplication in silk gland cells and silk gland growth probably by promoting protein synthesis and inhibiting protein degradation.

### The treatment with the ribosomal biogenesis pathway inhibitor Rbin-1 decreased DNA replication and silk protein synthesis in PSG cells

**Fig 6:**
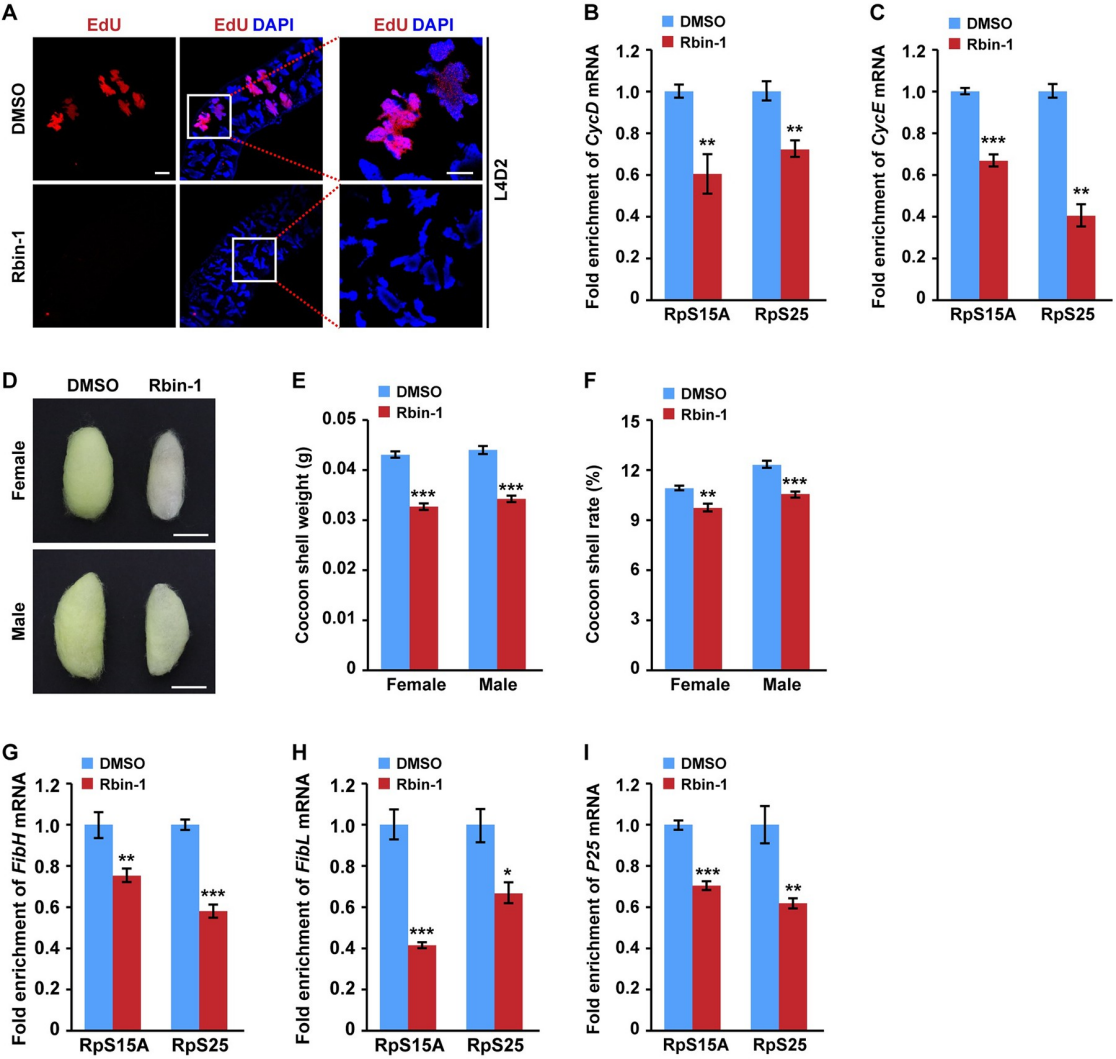
The treatment with the ribosome biogenesis pathway inhibitor Rbin-1 inhibits endoreplication and silk production. (**A**) EdU staining in silkworm PSG cells following treatment with Rbin-1 as an inhibitor of ribosome biogenesis. DMSO treatment was used as a control. The PSG cells of the control could be strongly stained with EdU, but no EdU signal was detected in PSG cells with Rbin-1 treatment. Scale bar, 100 μm. (**B-C**) Rbin-1 treatment decreased the translation level of *CycD* (B) and *CycE* (C). The anti-RpS15A and anti-RpS25 antibodies were used. (**D**) Changes in cocoon size of female and male silkworm larvae following Rbin-1 treatment. Scale bar, 1 cm. (**E-F**) Rbin-1 treatment decreased the cocoon shell weight (E) and cocoon shell rates (F). (**G-I**) Rbin-1 treatment decreased the translation level of *FibH* (G), *FibL* (H), and *P25* (I). Values were represented as means ±S.E. (error bars). For the significance test: *P < 0.05, **P < 0.01, and ***P < 0.001 versus the control.

Considering that PSG-specific *Fzr* mutation downregulated the ribosomal biogenesis pathway and blocked the translation of cyclin protein genes and PSG-specific silk protein genes, we next investigated the roles of ribosomal biogenesis pathway in regulating DNA replication and silk protein synthesis by using Rbin-1, an inhibitor that inhibits ribosome biogenesis [27]. First, we treated the silk glands from silkworm larvae at L4D2 with Rbin-1 inhibitor. Subsequent EdU staining showed that DNA synthesis was undergoing in PSG cells of the control, but this process was completely abrogated in PSG cells with Rbin-1 treatment (Fig 6A). Further TRAP experiment following RT-qPCR assay revealed that the mRNA translation of *CycD* and *CycE* in the PSG were obviously downregulated following Rbin-1 treatment (Fig 6B and 6C).

Next, we analyzed the effects of Rbin-1 treatment on silk production. Silkworm larvae at the fifth day of the fifth instar (L5D5) were injected by Rbin-1 inhibitor. The results showed that the cocoons of both female and male silkworm individuals with Rbin-1 treatment were smaller than that of the control (Fig 6D), and the cocoon shell weight and cocoon shell rates were decreased following Rbin-1 treatment (Fig 6E and 6F). Further analysis revealed that Rbin-1 treatment inhibited the mRNA translation of *FibH*, *FibL*, and *P25* (Fig 6G–6I). Taken together, our data indicated that Fzr mediates the ribosomal biogenesis pathway to promote DNA replication in endoreplicating silk gland cells and silk production.

## Discussion

Silkworm is an economically important insect and produces silk proteins for silk production in silk gland cells with endoreplication. Although several factors have been confirmed to regulate the endoreplication and growth of silk gland cells, the related regulatory mechanism remains largely unknown. Numerous evidences in other insects and mammals demonstrated that Fzr plays key roles in the endoreplication progression to regulate organ size by maintaining continuous DNA replication [7,18,23]. The present study in silkworm revealed that PSG-specific *Fzr* mutation disrupted PSG growth and DNA replication in PSG cells, suggesting that Fzr function is conserved in regulating endoreplication progression and organ growth in animals. In addition to the founding that the Fzr-H2Bub-Myc cascade in endoreplication, a cascade that we previously outlined [23], was conserved in silkworm PSG cells, we also found that *Fzr* mutation in PSG cells downregulated the transcription and translation of CycD and CycE, and induced a block of DNA replication and an arrest at G2/M phase. Collectively, our results, together with previous evidence, uncovers that Fzr precisely controls the entrance and maintenance of endoreplication in endocycling cells.

Intriguingly, we found that *Fzr* mutation impaired ribosome biogenesis pathway and increased ubiquitin-mediated protein degradation pathways. Ribosome functions a machine to synthesize proteins and ribosome biogenesis determines the ratio of protein translation [28–30]. Reducing ribosome biogenesis can cause an arrest of organ growth by repressing protein synthesis [31–33]. Our TRAP analysis showed that mRNA translation in PSG cells of *Fzr* mutant was highly inhibited and that the synthesis of silk proteins, including FibH, FibL, and P25, was reduced. Correspondingly, blocking the ribosome biogenesis using the Rbin-1 inhibitor also disrupted DNA replication and decreased silk production. In addition, most of DEPs involved in ubiquitin-mediated proteolysis and lysosome pathways were upregulated following PSG-specific *Fzr* mutation and an increase in the protein ubiquitination level was also detected. These observations suggest that Fzr may moderately protect protein degradation in PSG cells to maintain PSG growth, and *Fzr* mutation-caused decrease in silk production might be due to a repression in silk protein translation and/or an enhance in protein degradation. In the future, overexpressing key proteins of the ribosome pathway involved in protein translation and inhibiting the ubiquitin-mediated protein degradation will contribute to promote silk gland growth and elevate silk production.

Fzr-mediated APC/C E3 ubiquitin ligase activity is involved in the degradation of target proteins. To date, several cell cycle factors, such as CycB and Geminin, have been identified as the substrates of APC/C^Fzr^ [7,15,34,35]. Previous studies have reported that CycB acts as a key regulator to control the mitosis entry in mitotic cells and is absent in endocycling *Drosophila* salivary gland and silkworm silk gland cells [4,15,36–38]. Being consistent with this, our western blotting and proteome analysis also observed that CycB protein could not be detected in the endocycling PSG cells but was present again following *Fzr* mutation. In addition to CycB, we noted that 197 DEPs appeared after *Fzr* mutation compared with WT. Among these proteins, Kinetochore protein Spc25, Kinesin-like proteins, and other kinetochore related proteins are involved in spindle assembly, which is crucial for mitosis [39–41]. This indicate that Fzr may target and degrade more proteins involved in mitotic cell cycle to stop mitosis and induce the transition of mitosis-to-endoreplication. Further investigation on Fzr regulation of these potential targets will provide novel insights into molecular mechanism underlying endoreplication control.

## Materials and methods

### Insect strains

The non-diapaused silkworm strain D9L was used in the present study. Both the wild-type and transgenic silkworm stains were reared with fresh mulberry leaves at 25°C using an incubator with a 12 h light/12 h dark cycle.

### Recombinant plasmid construction and germ line transformation

A binary tissue specific CRISPR/Cas9 system was established to knockout the *Fzr* gene in silkworm posterior silk gland. The piggyBac-based plasmid pBac[3×*P3*-RFP, *FibH*-Cas9] (*FibH*-Cas9) was constructed to express Cas9 specifically in PSG under the control of silkworm PSG-specific *FibH* promoter with the red fluorescent protein (RFP) gene expression in the eyes under the control of the *3×P3* promoter. And the pBac[3×*P3*-EGFP, *U6*-*Fzr* gRNA] (*Fzr* gRNA) recombinant plasmid was generated to ubiquitously express the *Fzr* guide RNA (gRNA) driven by the silkworm *U6* promoter with the enhanced green fluorescent protein (EGFP) gene expressing in eyes. The gRNA targeting the second exon of silkworm *Fzr* gene was designed by the online webtool “CRISPR direct” (http://crispr.dbcls.jp/) [42]. By mixing the *FibH*-Cas9 and *Fzr* gRNA recombinant plasmid with a piggyBac helper plasmid separately, the mixtures were microinjected into non-diapaused fertilized eggs at preblastodermal stage for germ-line transformation. The microinjected eggs were cultured at 25°C with a humidity of 95%-100% until hatching and the offspring with expression of the marker gene in eyes were screened using a fluorescence microscopy. All related primers are listed in S6 Table.

### Detection of silkworm *Fzr* mutation

After crossing the *FibH*-Cas9 with *Fzr* gRNA transgenic strains, the PSG from the progeny with double fluorescence in eyes was dissected at different developing stage. After grinding the tissues into very fine powder with liquid nitrogen, the genomic DNA was isolated using the phenol/chloroform as previously described [43]. Following a PCR program using the genomic DNA as template and specific primers covering the mutant site of *Fzr* gene, the genomic PCR products were extracted and cloned into T-simple vector to sequence for evaluating the knockout efficiency. All related primers are listed in S6 Table.

### Western blotting

Total proteins were isolated from the silkworm silk gland and then quantified by the Bradford assay (Sigma) using a microplate reader (BioTek) at the absorbance of 562 nm. Equal amounts of total protein were subjected for Western blotting. The antibodies and dilutions used in the study were as follows: rabbit anti-Fzr (1:1000, Zoonbio Biotechnology), rabbit anti-CycB (1:1000, Zoonbio Biotechnology), mouse anti-CycD (1:1000, Zoonbio Biotechnology), mouse anti-CycE (1:1000, Zoonbio Biotechnology), rabbit anti-pH3 (1:1000, Thermo fisher), mouse anti-H2B (1:10, 000; Beyotime), rabbit anti-H2Bub (1:20, 000; Cell Signaling), rabbit anti-Ub (1:1000, Proteintech), rabbit anti-RpS15A (1:5000, Abclonal), rabbit anti-RpS25 (1:5000, Abclonal), rabbit anti-GNS (1:1000, Sangon Biotech), rabbit anti-UBE2C (1:1000, Sangon Biotech) and mouse anti-Tubulin (1:10000, Beyotime).

### Quantitative RT-PCR (RT-qPCR) and RT-PCR

Total RNA was extracted from the silkworm silk gland at different developing stage using the Trizol reagent (Invitrogen), as described previously [44]. 2 μg total RNA was used for synthesizing cDNA templates in a 20 μl reaction mixture with the M-MLV Reverse Transcriptase Kit (Promega). RT-qPCR assays in three replicates were performed with a SYBR Premix ExTaq Kit (TAKARA) on a qTower 2.2 Real-time PCR Detection System (Jena). The silkworm ribosomal protein L3 (*RpL3*) gene were used as internal control. The relative mRNA expression level of each gene was calculated using the ΔΔCT method. Gel electrophoresis-based semiquantitative RT-PCR examination was used to detect the transcription level of *Fzr* and *CycB* in silk gland. The silkworm *Actin* gene was used as the internal control. All related primers are listed in S6 Table.

### EdU staining

EdU staining was performed as previously described [23]. Briefly, the silkworm silk gland was dissected at the second day of the second larval instar (L2D2) and L4D2, and then cultured with 100 μg/mL EdU according to the manufacturer’s protocol for Cell Light EdU Apollo 567 *in vitro* Kit (Ribobio). After culturing for 2 h at room temperature, the glands were fixed with 4% paraformaldehyde for 30 min. Following a three-time washing with 25 mM glycine, the samples were successively stained with Apollo dye for 30 min and DAPI for 30 min, and then mounted in Vectashield buffer. Fluorescence signals were captured by confocal microscopy (Zeiss LSM 880 and Olympus Fv1000).

### DNA quantification

3–5 PSGs (about 500 cells per PSG) from silkworm larvae at L4D2 and 1×10^7^ cultured BmE cells were separately collected and subsequently lysed in DNA SDS lysis-phenol buffer supplemented with proteinase K. After a digestion with RNAase, total genomic DNA were extracted and purified. DNA content of each sample was spectrophotometrically quantified at OD 260 nm using an Agilent 2100 Bioanalyzer System (Agilent, Palo Alto, CA, USA). Based on the 2C genome of diploid BmE cells as a reference, the genome content of PSG cells was calculated.

### Protein extraction and LC-MS/MC analysis

For LC-MS/MS analysis, total protein in silk gland was extracted using the SDT buffer (4% SDS, 100 mM Tris-HCl, 1mM DTT, pH7.6) and quantified by the Bradford assay (Sigma) at the absorbance of 562 nm using a microplate reader (BioTek). Then, the extracted protein was digested with trypsin as previously described [45]. Each protein sample was subjected to LC-MS/MS analysis using the label-free quantitative proteomic approach by Shanghai Applied Protein Technology (Shanghai, China).

All raw data for each sample from LC-MS/MS were searched using the MaxQuant 1.5.3.17 software with a false discovery rate (FDR) of 1% for identification and quantitation analysis against silkworm database (uniprot_*Bombyx_mori*_18486_20201203.fasta). Statistical significance of the difference between groups was evaluated using Student’s t-test, and p value < 0.05 and log2 fold change (log2FC) ≥ 1.0 was set as a criterion. Based on the software program Blast2GO, gene ontology (GO) terms of DEPs were mapped and the GO annotation results were plotted by R scripts. The online Kyoto Encyclopedia of Genes and Genomes (KEGG) database (http://geneontology.org/) was used for pathway enrichment analysis.

### Translating ribosome affinity purification (TRAP) analysis

According to the principle of the TRAP approach [46,47], PSGs in both WT and *Fzr* mutant animals were collected at L4D2. A copy of PSGs samples cultured in cycloheximide (Sigma) for 2 h and then lysed in NP-40 lysis buffer containing RNase inhibitor RNasin (Promega). The ribosome-mRNA complexes were purified based on Co-IP assay with specific antibody against RpS15A and RpS25, and the mRNAs in the IP products were then isolated for further RT-qPCR examination. Another copy of PSGs samples were used to isolate total mRNA, which was used as the internal control in RT-qPCR analysis. The mRNA amount was quantified using an Agilent 2100 Bioanalyzer System (Agilent, Palo Alto, CA, USA).

### Rbin-1 inhibitor treatment

The Rbin-1 inhibitor (MedChemExpress) was used for blocking ribosome biogenesis as described previously [27]. Briefly, the silkworm silk glands were dissected at L4D2, and then cultured with 2 μM Rbin-1 for 6 h at room temperature. After the drug treatment, a set of glands were used for EdU staining. Another set of glands were collected for TRAP experiment to detect the translation level of *CycD* and *CycE*. In addition, silkworm larvae at L5D5 were injected with Rbin-1 (2 μg per larva). At 12 h after injection, silk glands from six silkworm individuals were isolated for further TRAP experiments to analyze the translation level of *FibH*, *FibL*, and *P25*. The remaining silkworm individuals were used to evaluate silk production at the pupal stage. DMSO treatment was used as a control.

### Statistical analysis

Data from three independent biological replicates are presented as the mean ± SE. Statistical significance (*P*-value) was evaluated by an unpaired, two-tailed Student’s t-test and denoted as follows: *P < 0.05, **P < 0.01, and ***P < 0.001 versus the control.

## Supporting information

S1 Fig *Fzr* mutation abrogates PSG growth during silkworm development. (**A**) Transgenic Cas9 protein was specifically expressed in PSG cells. (**B-E**) The size of PSG from silkworm larvae at L2 (B), L3 (C), L4D2 (D), and L5D3 (E). L2, the second larval instar; L3, the third larval instar; L4D2, the second day of the fourth larval instar; L5D3, the third day of the fifth larval instar. Scale bar for L2 and L3, 2 mm; Scale bar for L4D2 and L5D3, 1 cm.
(TIF)

S2 Fig EdU staining of DNA replication and cell number quantification. (**A**) DNA replication was not changed in PSG cells at L2D2. L2D2, the second day of the second larval instar. (**B-C**) PSG-specific *Fzr* mutation had no effect on DNA replication of ASG (B) and MSG (C) cells. L4D2, the second day of the fourth larval instar. ASG, anterior silk gland. MSG, middle silk gland. (**D**) *Fzr* mutation had no effect on the number of PSG cells.
(TIF)

S3 Fig Expression profile of cell cycle-related genes in the PSG during silkworm development. (**A**) *Fzr* was continuously expressed in the PSG during the fourth larval instar, while *CycB* was not expressed. The *Actin* gene was used as the internal control. L4D0, just the fourth larval instar; L4D1, the first day of the fourth larval instar; L4D2, the second day of the fourth larval instar; L4D3, the third day of the fourth larval instar. (**B**) PSG-specific *Fzr* mutation promoted *CycB* transcription. (**C-E**) *Fzr* mutation decreased the ubiquitinated level of H2B protein (C), *Myc* transcription (D), and the transcription of the *MCM* genes (E) in the PSG. Values were represented as means ±S.E. (error bars). For the significance test: *P < 0.05, **P < 0.01, and ***P < 0.001 versus the control. WT, wild type.
(TIF)

S4 Fig Numbers of spectrums, peptides and the associated proteins were identified in all samples. (TIF)

S5 Fig Clustering analysis and GO annotation of DEPs. (**A**) Hierarchical clustering of all DEPs. Red, proteins with high expression levels; Blue, proteins with low expression levels. (**B**) GO annotation of molecular function class. Size of dots represented numbers of enriched proteins; Color of dots represented the GO cluster with a highlighted representative term.
(TIF)

S6 Fig TRAP analysis following RT-qPCR. The translation level of silk protein genes (A-C) and cyclin proteins (D-E) were decreased following *Fzr* mutation. The anti-RpS15A and anti-RpS25 antibodies were used. For the significance test: *P < 0.05, **P < 0.01, and ***P < 0.001 versus the control. WT, wild type.
(TIF)

S1 Table The list of identified peptides. (XLSX)

S2 Table The list of identified proteins. (XLSX)

S3 Table The list of the all the DEGs. (XLSX)

S4 Table The list of the DEGs that classified in biological process and molecular function. (XLSX)

S5 Table The enriched KEGG pathways. (XLSX)

S6 Table Primers used in the present study. (DOCX)
