# Supplementary figures and images for: Fzr regulates silk gland growth by promoting endoreplication and protein synthesis in the silkworm
Source: PLoS Genet. 2023 Jan 18;19(1):e1010602. doi: 10.1371/journal.pgen.1010602 (PMC9886304; doi:10.1371/journal.pgen.1010602)

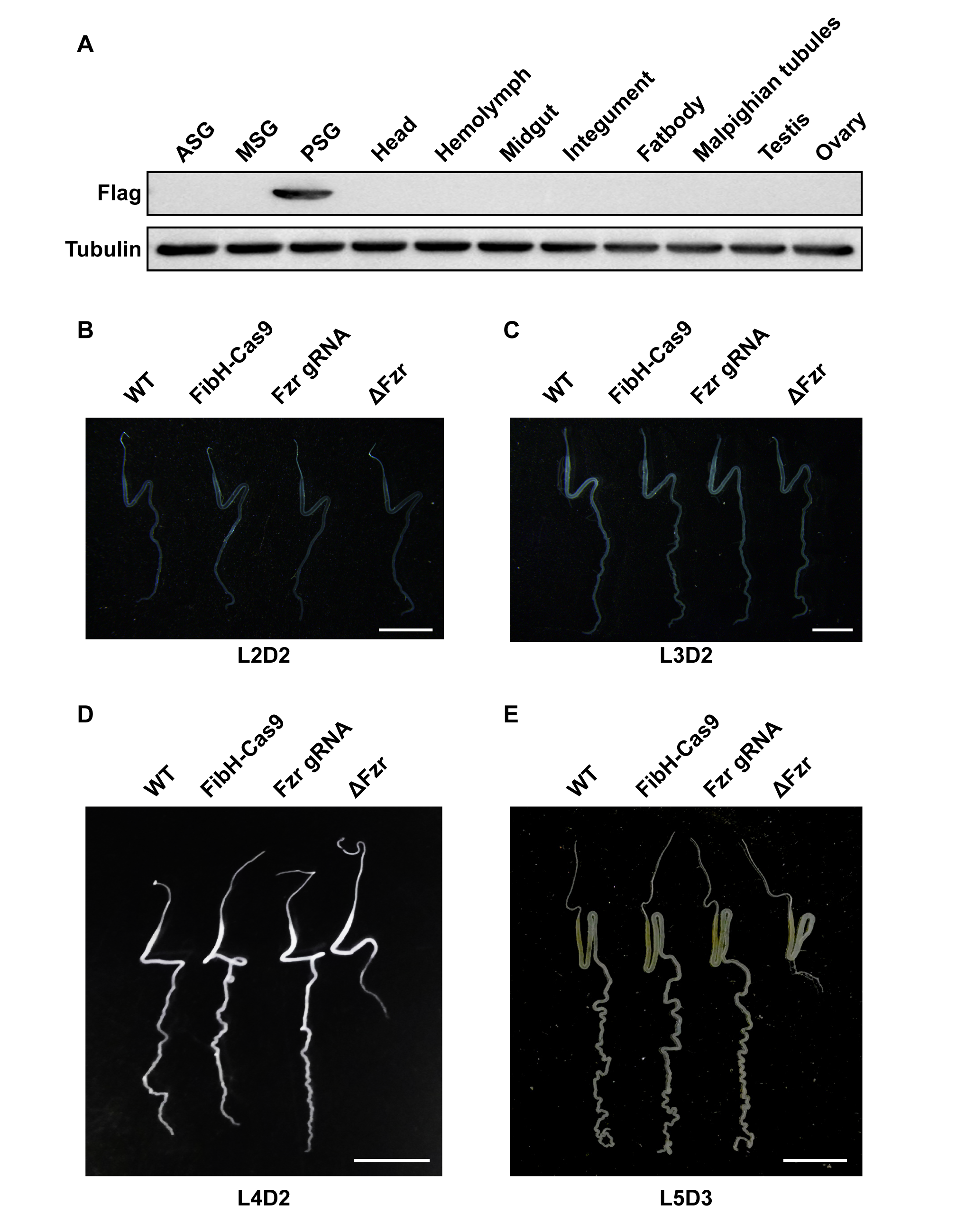

Supplement: S1 Fig — (A) Transgenic Cas9 protein was specifically expressed in PSG cells. (B-E) The size of PSG from silkworm larvae at L2 (B), L3 (C), L4D2 (D), and L5D3 (E). L2, the second larval instar; L3, the third larval instar; L4D2, the second day of the fourth larval instar; L5D3, the third day of the fifth larval instar. Scale bar for L2 and L3, 2 mm; Scale bar for L4D2 and L5D3, 1 cm. (TIF) [file pgen.1010602.s001.tif]

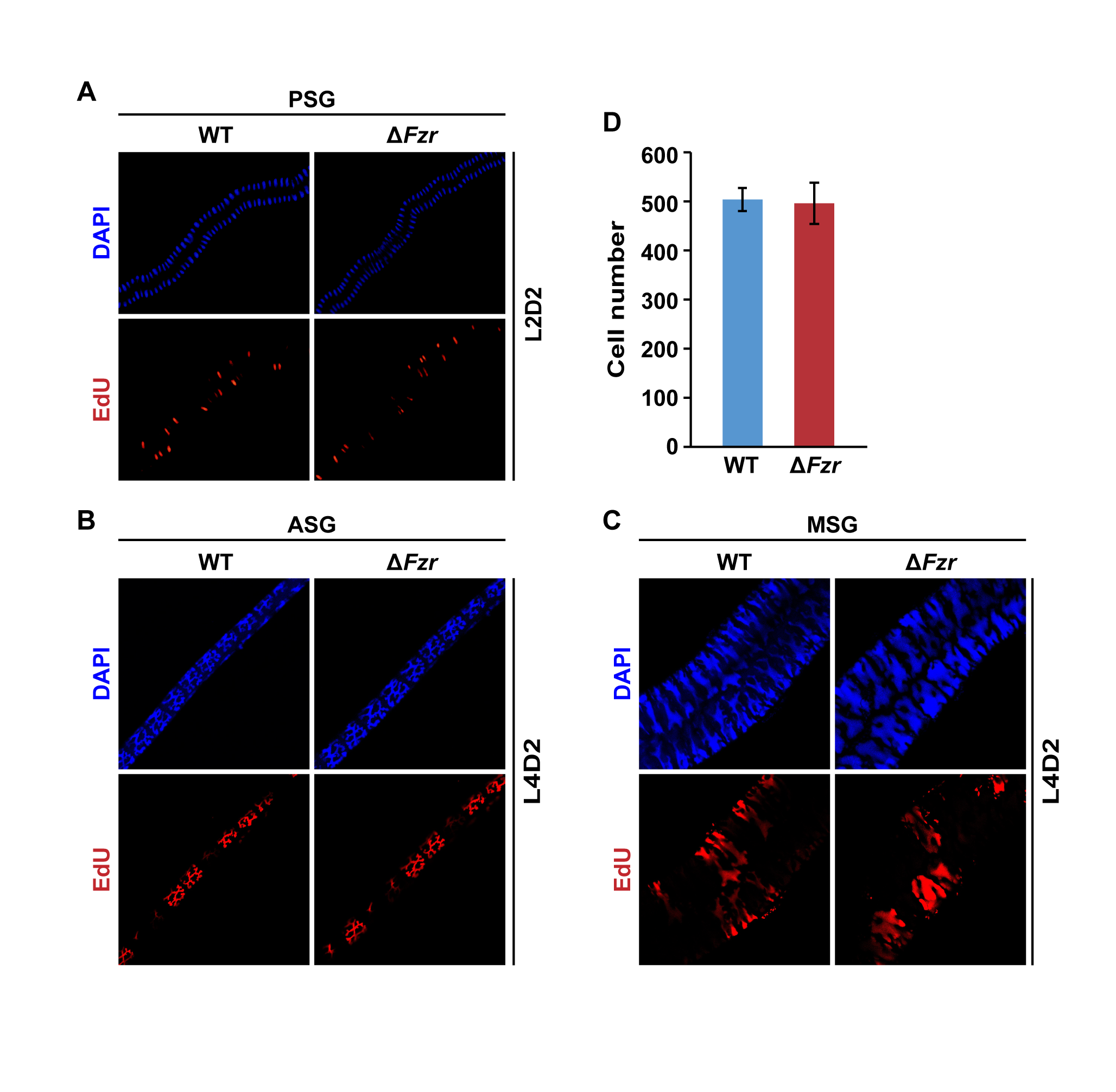

Supplement: S2 Fig — (A) DNA replication was not changed in PSG cells at L2D2. L2D2, the second day of the second larval instar. (B-C) PSG-specific Fzr mutation had no effect on DNA replication of ASG (B) and MSG (C) cells. L4D2, the second day of the fourth larval instar. ASG, anterior silk gland. MSG, middle silk gland. (D) Fzr mutation had no effect on the number of PSG cells. (TIF) [file pgen.1010602.s002.tif]

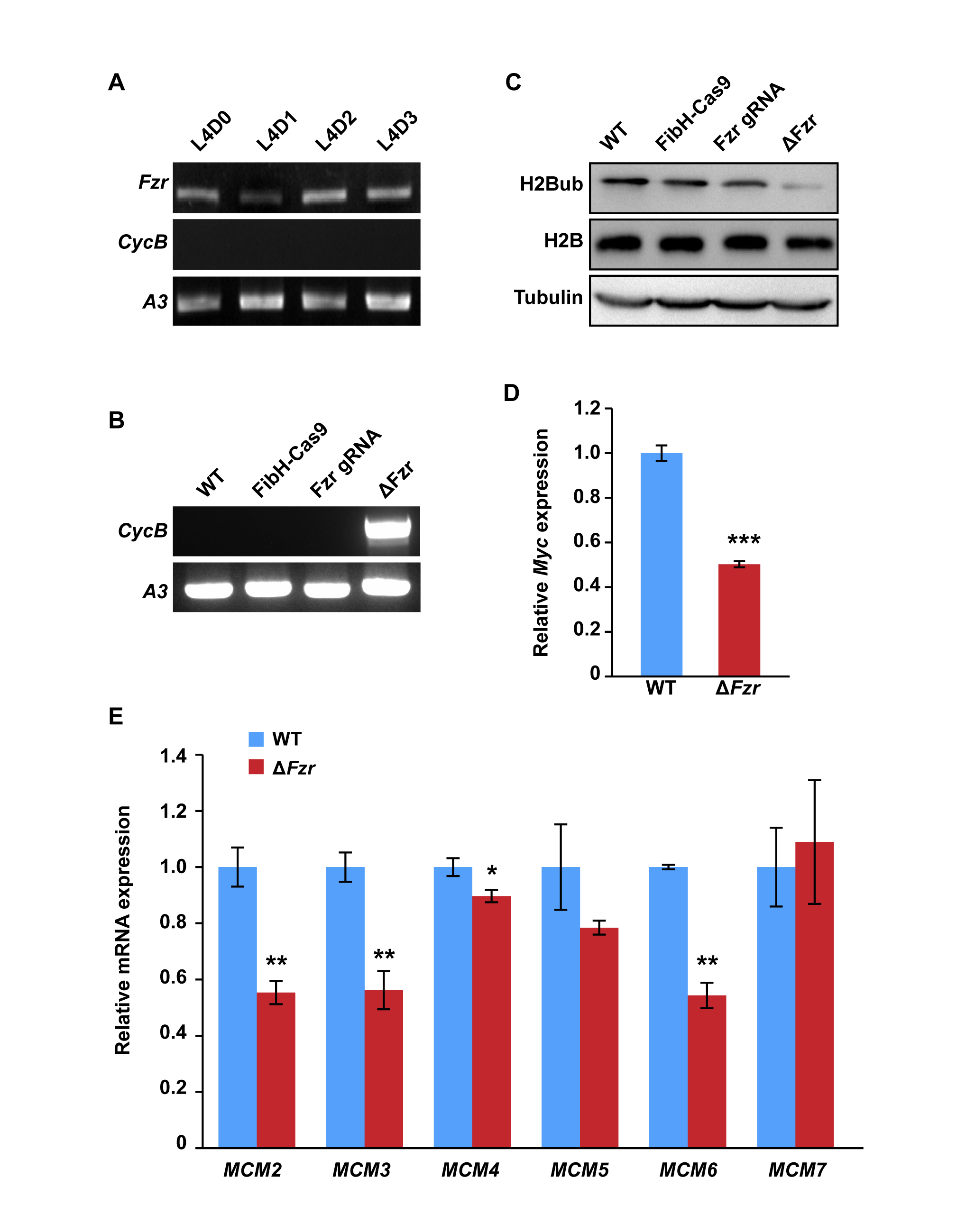

Supplement: S3 Fig — (A) Fzr was continuously expressed in the PSG during the fourth larval instar, while CycB was not expressed. The Actin gene was used as the internal control. L4D0, just the fourth larval instar; L4D1, the first day of the fourth larval instar; L4D2, the second day of the fourth larval instar; L4D3, the third day of the fourth larval instar. (B) PSG-specific Fzr mutation promoted CycB transcription. (C-E) Fzr mutation decreased the ubiquitinated level of H2B protein (C), Myc transcription (D), and the transcription of the MCM genes (E) in the PSG. Values were represented as means ±S.E. (error bars). For the significance test: *P < 0.05, **P < 0.01, and ***P < 0.001 versus the control. WT, wild type. (TIF) [file pgen.1010602.s003.tif]

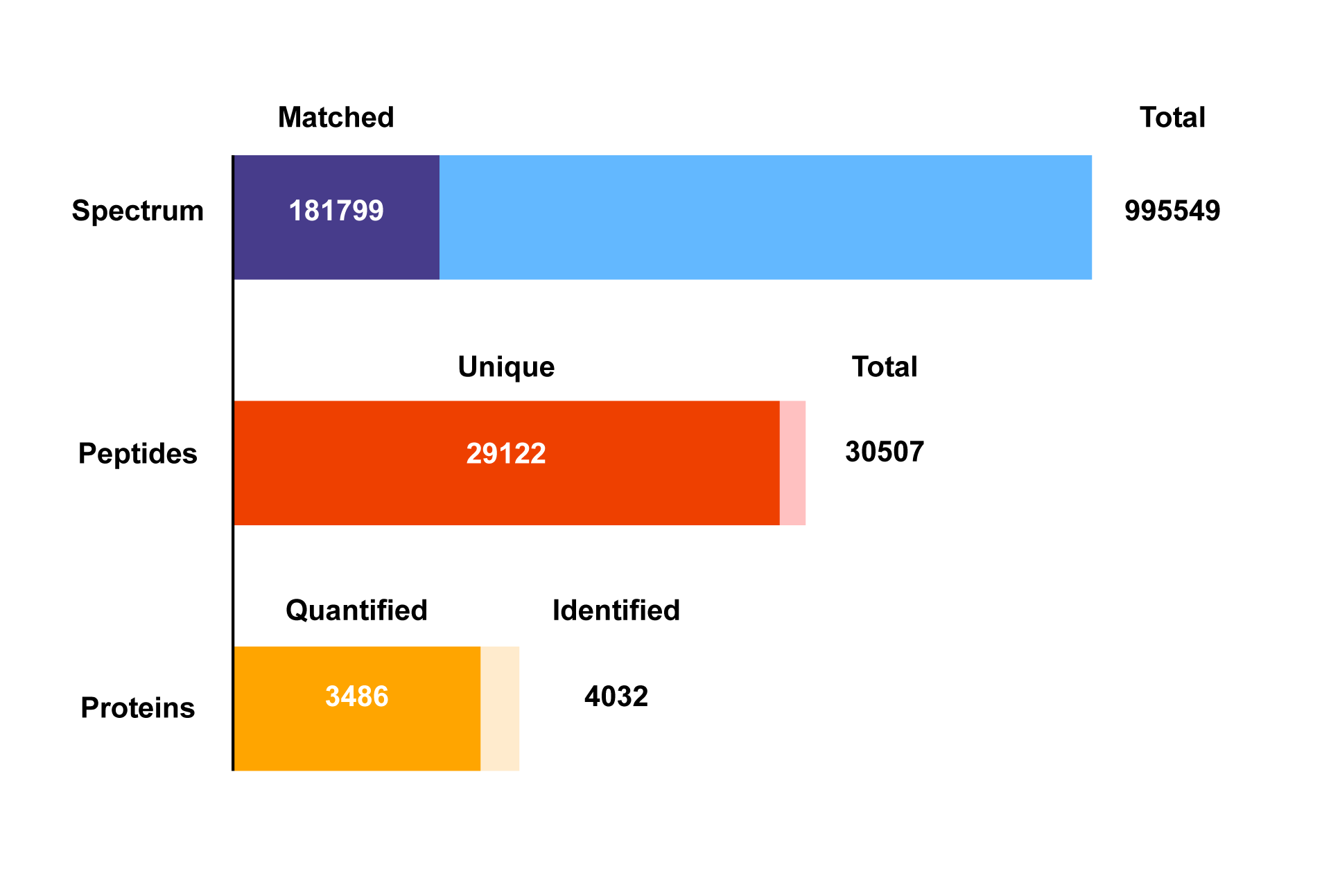

Supplement: S4 Fig — (TIF) [file pgen.1010602.s004.tif]

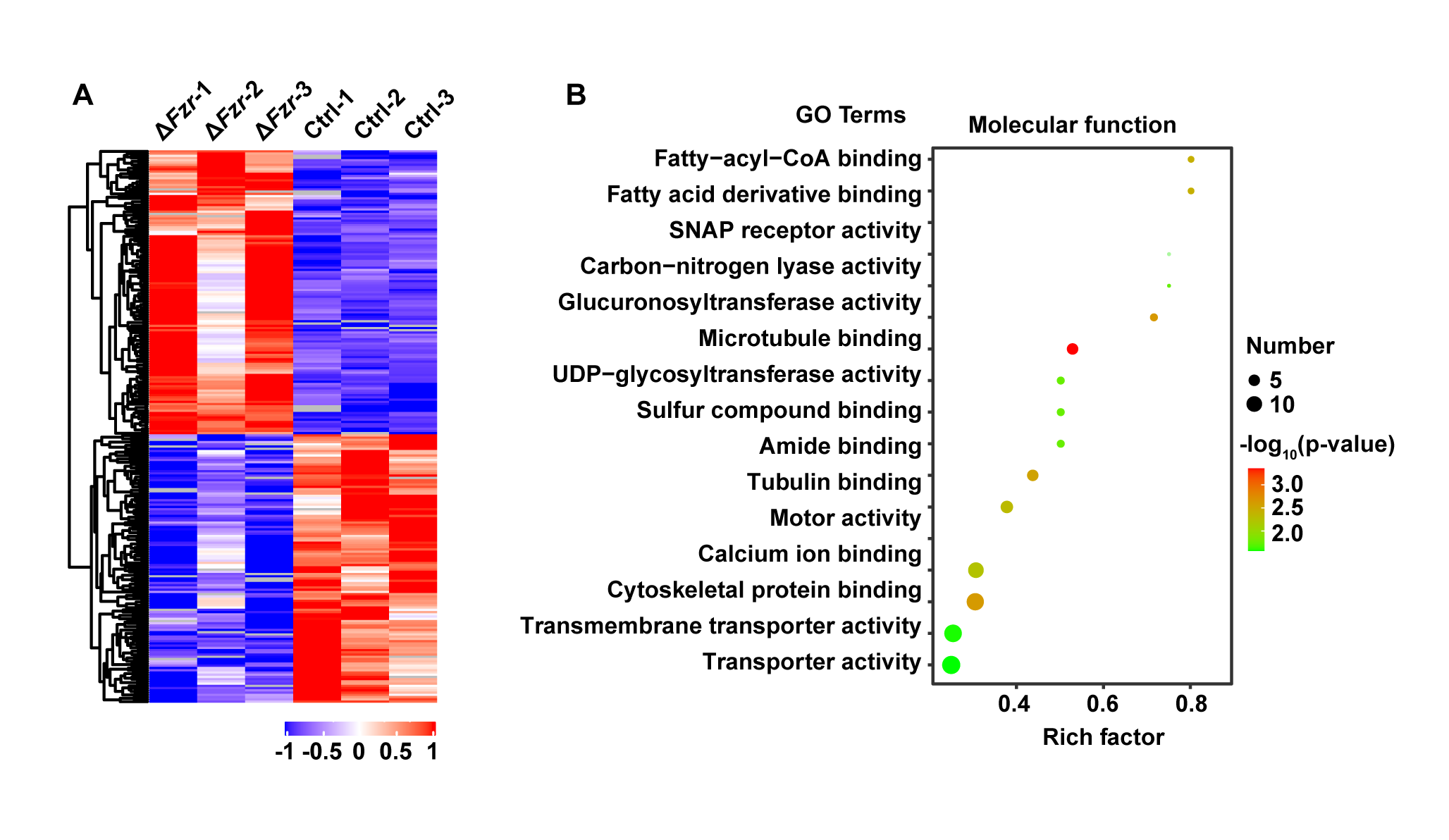

Supplement: S5 Fig — (A) Hierarchical clustering of all DEPs. Red, proteins with high expression levels; Blue, proteins with low expression levels. (B) GO annotation of molecular function class. Size of dots represented numbers of enriched proteins; Color of dots represented the GO cluster with a highlighted representative term. (TIF) [file pgen.1010602.s005.tif]

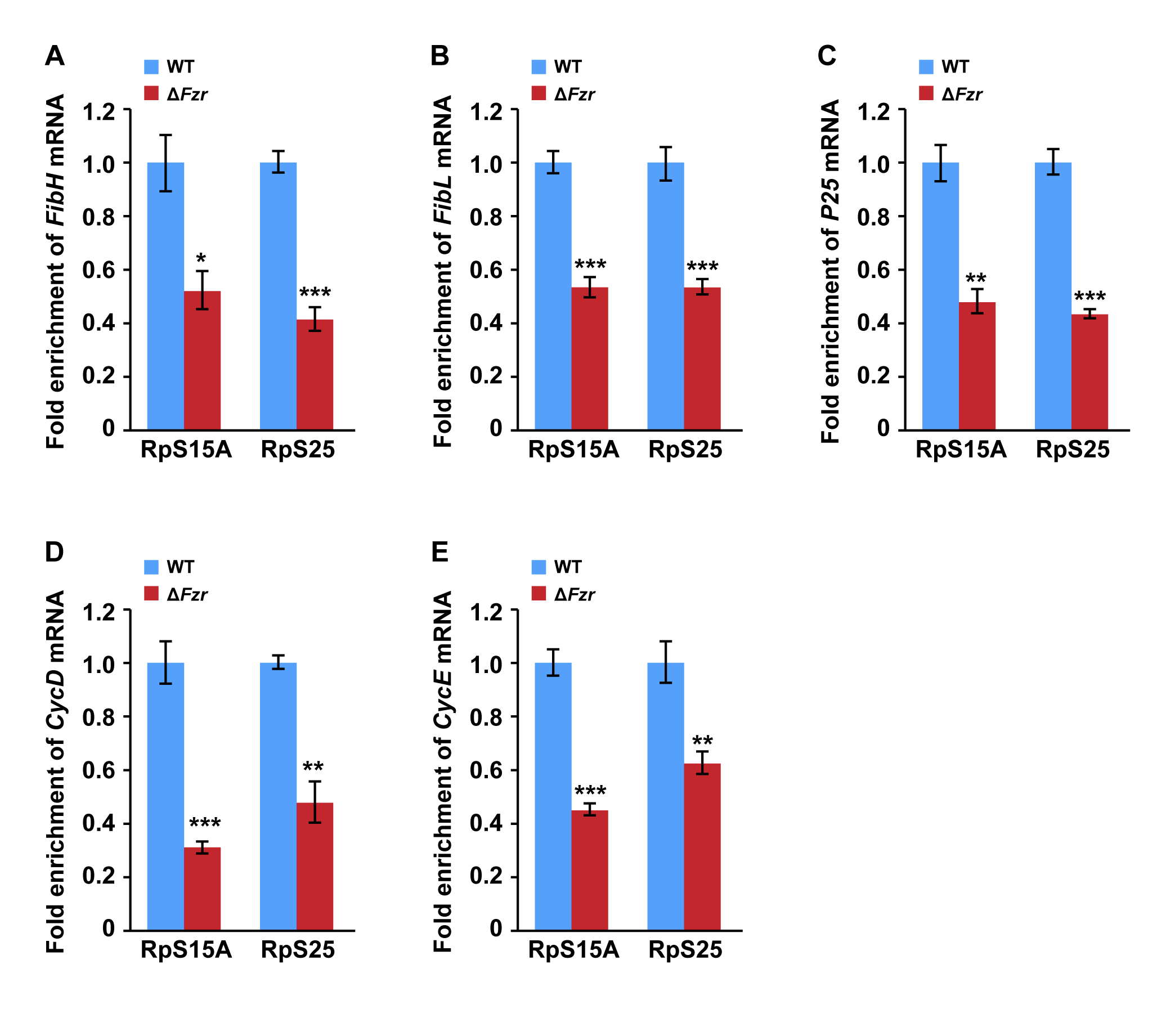

Supplement: S6 Fig — The translation level of silk protein genes (A-C) and cyclin proteins (D-E) were decreased following Fzr mutation. The anti-RpS15A and anti-RpS25 antibodies were used. For the significance test: *P < 0.05, **P < 0.01, and ***P < 0.001 versus the control. WT, wild type. (TIF) [file pgen.1010602.s006.tif]
