# Supplementary material for: Fzr regulates silk gland growth by promoting endoreplication and protein synthesis in the silkworm
Source: PLoS Genet. 2023 Jan 18;19(1):e1010602. doi: 10.1371/journal.pgen.1010602 (PMC9886304; doi:10.1371/journal.pgen.1010602)
Supplement: S6 Table — (DOCX) [file pgen.1010602.s012.docx]

**S6 Table. Primers used in the present study**

| Purpose | Primer name | Primer sequence |
| --- | --- | --- |
| Construction of recombinant plasmid | *FibH* promoter | Forward: 5' CCGCTCGAGTAATTAGGTAGTGTTTAAGCTTGTTG 3' |
|  |  | Reverse: 5' GGACTAGTGAGAGTTGGAACCGAACTG 3' |
|  | *Fzr* gRNA | Forward: 5' AAGTGTGGGGACCGGAGAAGCTTCT 3' |
|  |  | Reverse: 5' AAACAGAAGCTTCTCCGGTCCCCAC 3' |
|  | *Ser-*PA | Forward: 5' CGCGGATCCTACAACTAAACACGACTTGGAGT 3' |
|  |  | Reverse: 5' CGACGCGTTTCGTCAATGTATCAGTTTTGGT 3' |
| RT-PCR | *Fzr* | Forward: 5' AAGGCGACCCGCAAGATA 3' |
|  |  | Reverse: 5' CCTCCTGAAGCCAACGATT 3' |
|  | *CycB* | Forward: 5' GGCACAGATTCAAGACCAAGA 3' |
|  |  | Reverse: 5' TGTTTTCCGTAAAGAGTCAGTTC 3' |
| RT-qPCR | *MCM2* | Forward: 5' GATGAAAATGCCGATGCC 3' |
|  |  | Reverse: 5' AACTCAACATGAAAGGATGC 3' |
|  | *MCM3* | Forward: 5' CAGCCGAATCCATTTACG 3' |
|  |  | Reverse: 5' CTTGCCATGTCGCTATCC 3' |
|  | *MCM4* | Forward: 5' TGATATAAGTCTGATGCGGGA 3' |
|  |  | Reverse: 5' AGTTCAACAACGGAAGAAAGTC 3' |
|  | *MCM5* | Forward: 5' GTTTTGGCTGATGGTGGT 3' |
|  |  | Reverse: 5' CGAGAATTTAATGTAGTGGTGA 3' |
|  | *MCM6* | Forward: 5' AGATACGAATCCAGGAAACCC 3' |
|  |  | Reverse: 5' CACATCAGGCACTACAATCAG 3' |
|  | *MCM7* | Forward: 5' TAGTCTTGCCCCCGAAAT 3' |
|  |  | Reverse: 5' CAAAACAGCAGCAGTGAGG 3' |
|  | *Myc* | Forward: 5' GAAGAGACGCACGAAGATCC 3' |
|  |  | Reverse: 5' AGGGTGGTGTGTCAGGTCTC 3' |
|  | *FibH* | Forward: 5' TCTGTGTCATCTGCTTCATCTCG 3' |
|  |  | Reverse: 5' TATCCAGGACGAAGTAAGAAACAA 3' |
|  | *FibL* | Forward: 5' ATACCGATTGGTCACATAACAG 3' |
|  |  | Reverse: 5' GCAGATAGATGGGCGATAA 3' |
|  | *P25* | Forward: 5' AGCCGCTGTGGCAGTTTTG 3' |
|  |  | Reverse: 5' TAGGTGGCGTTGAAGTATGG 3' |
|  | *CycD* | Forward: 5' GCGTTTGACTTCGTGGAACC 3' |
|  |  | Reverse: 5' CGTTGACAGACATTCGCACG 3' |
|  | *CycE* | Forward: 5' CCCAAGACAATCCAGGCAA 3' |
|  |  | Reverse: 5' AGAGGCGAGTCCACCCCA 3' |
